# α-Ketoglutarate improves cardiac insufficiency through NAD^+^-SIRT1 signaling-mediated mitophagy and ferroptosis in pressure overload-induced mice

**DOI:** 10.1186/s10020-024-00783-1

**Published:** 2024-01-22

**Authors:** Hao Yu, Daojing Gan, Zhen Luo, Qilin Yang, Dongqi An, Hao Zhang, Yingchun Hu, Zhuang Ma, Qingchun Zeng, Dingli Xu, Hao Ren

**Affiliations:** 1 State Key Laboratory of Organ Failure Research, Department of Cardiology, Nanfang Hospital, Southern Medical University, 1838 Northern Guangzhou Ave, Guangzhou, Guangdong 510515 China; 2 Key Laboratory for Organ Failure Research, Ministry of Education of the People’s Republic of China, 1838 Northern Guangzhou Ave, Guangzhou, Guangdong 510515 China; 3 Department of Cardiovascular Surgery, Nanfang Hospital, Southern Medical University, 1838 Northern Guangzhou Ave, Guangzhou, Guangdong 510515 China; 4 Department of Rheumatology, Nanfang Hospital, Southern Medical University, 1838 Northern Guangzhou Ave, Guangzhou, Guangdong 510515 China

**Keywords:** α-Ketoglutarate, Cardiac insufficiency, Mitophagy, Ferroptosis, Transverse aortic constriction, Angiotensin II, NAD, SIRT1

## Abstract

**Background:**

In heart failure (HF), mitochondrial dysfunction and metabolic remodeling lead to a reduction in energy productivity and aggravate cardiomyocyte injury. Supplementation with α-ketoglutarate (AKG) alleviated myocardial hypertrophy and fibrosis in mice with HF and improved cardiac insufficiency. However, the myocardial protective mechanism of AKG remains unclear. We verified the hypothesis that AKG improves mitochondrial function by upregulating NAD^+^ levels and activating silent information regulator 2 homolog 1 (SIRT1) in cardiomyocytes.

**Methods:**

In vivo, 2% AKG was added to the drinking water of mice undergoing transverse aortic constriction (TAC) surgery. Echocardiography and biopsy were performed to evaluate cardiac function and pathological changes. Myocardial metabolomics was analyzed by liquid chromatography‒mass spectrometry (LC‒MS/MS) at 8 weeks after surgery. In vitro, the expression of SIRT1 or PINK1 proteins was inhibited by selective inhibitors and siRNA in cardiomyocytes stimulated with angiotensin II (AngII) and AKG. NAD^+^ levels were detected using an NAD test kit. Mitophagy and ferroptosis levels were evaluated by Western blotting, qPCR, JC-1 staining and lipid peroxidation analysis.

**Results:**

AKG supplementation after TAC surgery could alleviate myocardial hypertrophy and fibrosis and improve cardiac function in mice. Metabolites of the malate-aspartate shuttle (MAS) were increased, but the TCA cycle and fatty acid metabolism pathway could be inhibited in the myocardium of TAC mice after AKG supplementation. Decreased NAD^+^ levels and SIRT1 protein expression were observed in heart of mice and AngII-treated cardiomyocytes. After AKG treatment, these changes were reversed, and increased mitophagy, inhibited ferroptosis, and alleviated damage in cardiomyocytes were observed. When the expression of SIRT1 was inhibited by a selective inhibitor and siRNA, the protective effect of AKG was suppressed.

**Conclusion:**

Supplementation with AKG can improve myocardial hypertrophy, fibrosis and chronic cardiac insufficiency caused by pressure overload. By increasing the level of NAD^+^, the SIRT-PINK1 and SIRT1-GPX4 signaling pathways are activated to promote mitophagy and inhibit ferroptosis in cardiomyocytes, which ultimately alleviates cardiomyocyte damage.

**Supplementary Information:**

The online version contains supplementary material available at 10.1186/s10020-024-00783-1.

## Background

Heart failure (HF) is a severe manifestation or advanced stage of a variety of heart diseases with multiple pathophysiological features, including mitochondrial damage and dysfunction (Weiss et al. 2005; McDonagh et al. 2021). Metabolic remodeling occurs in cardiomyocytes in HF, leading to the inhibition of mitochondrial oxidative phosphorylation, a reduction in adenosine triphosphate (ATP) production and myocardial systolic and diastolic dysfunction (van Bilsen et al. 2004; Lopaschuk et al. 2021). The factors released by mitochondrial damage can mediate inflammation and oxidative stress, which further aggravate cell damage and energy metabolism (Lopaschuk et al. 2021). Therefore, reducing mitochondrial injury is essential for protecting cardiomyocytes and improving energy metabolism.

It has been found that supplementation with AKG, an intermediate of tricarboxylic acid (TCA) cycle, can alleviate diseases mediated by mitochondrial damage and oxidative stress by promoting mitophagy (Asadi Shahmirzadi et al. 2020; Wang et al. 2020; Bayliak and Lushchak 2021; Liu et al. 2023). Mitophagy is a self-protective process in which cells remove damaged mitochondria. AKG supplementation can promote mitophagy in rat cartilage tissue and alleviate IL-1β-induced osteoarthritis (Liu et al. 2023). In our previous study, AKG promoted mitophagy and reduced cardiac disfunction in mice with HF (An et al. 2021). However, the mechanism by which AKG affects mitophagy remains unclear.

In addition to the TCA cycle, AKG is also involved in several metabolic processes, such as the malate-aspartate shuttle (MAS) (Gevi et al. 2017). As a substrate, AKG participates in the mutual conversion of malic acid and oxaloacetic acid, oxidizes NADH to NAD^+^, and transports malic acid and NAD^+^ to mitochondria to participate in the TCA cycle (Borst 2020). Decreased NAD^+^ and silent information regulator 2 homolog 1 (SIRT1) levels have been observed in cardiomyocyte cardiomyopathy or HF caused by mitochondrial dysfunction (Horton et al. 2016; Lee et al. 2016). SIRT1 is a deacetylase and dependent on the level of NAD^+^ (Mouchiroud et al. 2013). SIRT1 can regulate autophagy by PINK1-Parkin pathway (Jiang et al. 2021). In addition, SIRT1 regulates ferroptosis by the GPX4-related pathway, which is important in cardiomyocyte damage (Stockwell et al. 2017; Ma et al. 2020; Li et al. 2021). Therefore, we speculate that the regulation of NAD^+^-related pathways may be the potential mechanism by which AKG protects cardiomyocytes.

Therefore, we attempted to verify that AKG protects cardiomyocytes through the NAD^+^/SIRT1 pathway. This study aimed to (1) determine the effects of AKG on myocardial metabolomics, (2) identify SIRT1 as a factor that improves the effect of AKG on cardiomyocyte injury, and (3) explore the role of SIRT1-related pathways in AKG-mediated cardiomyocyte protection.

## Materials and methods

### Experimental animals

**Fig. 1:**
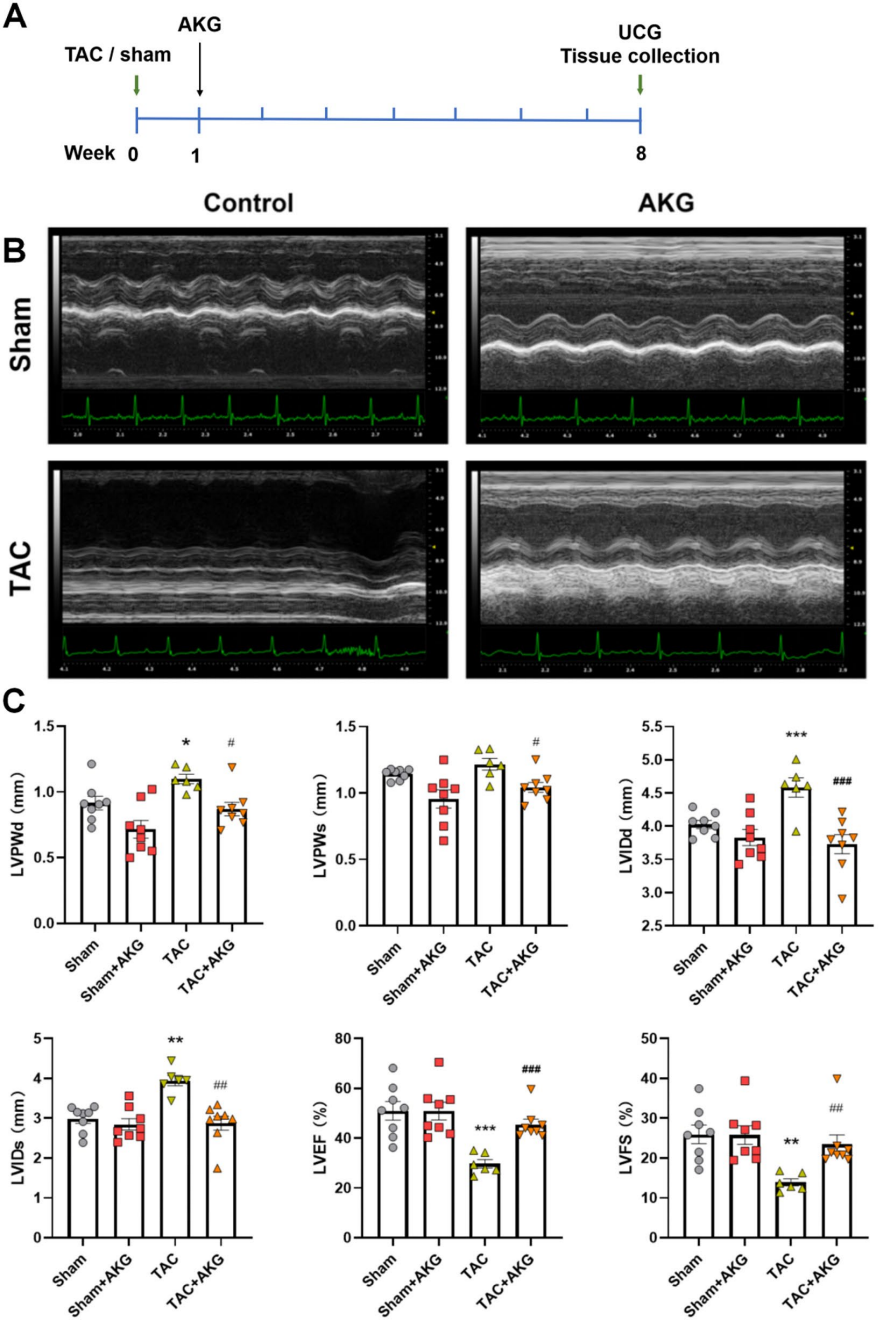
AKG improves cardiac dysfunction in TAC mice. (**A**) The timeline of vivo experiments. (**B**) Echocardiogram of each group at 8 weeks after surgery. (**C**) Statistical analysis of left ventricular posterior wall thickness at diastole (LVPWd), left ventricular posterior wall thickness at systole (LVPWs), left ventricular internal diameter at diastole (LVIDd), left ventricular internal diameter at systole (LVIDs), left ventricular ejection fraction (LVEF), and left ventricular shortening fraction (LVFS). *P < 0.05, **P < 0.01, ***P < 0.001 vs. Sham; ^#^*P* < 0.05, ^##^*P* < 0.01, ^###^*P* < 0.001 vs. TAC

All C57BL/6J mice and newborn Sprague‒Dawley (SD) rats were purchased from the Experimental Animal Center of Southern Medical University. Mice were housed on a natural light/dark cycle and fed a regular mouse diet. Eight-week-old male mice were subjected to transverse aortic constriction (TAC) surgery or sham surgery according to previously report (Liu et al. 2019). After surgery, mice were divided into four groups randomly: (1) Sham group; (2) TAC group; (3) Sham mice treated with AKG; (4) TAC mice treated with AKG. 2% AKG (Aladdin, USA) was added to the drinking water of mice in AKG treatment group. At 8 weeks after surgery, mice were subjected to echocardiography (Vevo2100,VisualSonics, Canada) with a 21-MHz transducer (MS400). Then the mice were killed by excessive anesthesia and heart tissues were collected. Figure 1A showed the timeline of treatment.

### Isolation and culture of neonatal rat ventricular myocytes (NRVMs)

NRVMs were isolated cultivated as previously described (Liu et al. 2019). Cardiomyocytes were stimulated with 2 µM AngII (Abcam, USA) and treated with 2 µM AKG (Aladdin, USA) for 24 h(An et al. 2021).

### Cardiomyocyte protein expression intervention

#### siRNA transfection

To knock down SIRT1 and PINK1, cardiomyocytes (60–70% confluence) in 6-well plates were incubated with a mixture of SIRT1, PINK1 or scrambled siRNA (60 nmol/L) (RiboBio, China), Lipofectamine 3000 (Invitrogen, USA), Opti-MEM (Gibco, USA) and basic medium (containing 3% FBS) for 6–8 h and then incubated in complete medium (containing 10% FBS and antibiotics) for 48 h.

#### Selective SIRT1 inhibitors

EX527 (Selleck Chemicals, USA) was dissolved in DMSO. According to the results of the preliminary experiment, cardiomyocytes were stimulated with 50 µM EX527 for 12 h before being treated.

### Heart tissue sectioning and staining

Heart tissues were sectioned and stained with hematoxylin-eosin (HE) (Servicebio, China), Masson (Servicebio, China) and wheat germ agglutinin (WGA) (Sigma, USA) according to the manufacturers’ protocols. The images were analyzed with ImageJ software (NIH).

### Untargeted metabolomics

We analyzed samples from hearts by liquid chromatography‒mass spectrometry (LC‒MS) with a Thermo Ultimate 3000 (Thermo Fisher Scientific, USA) and TripleTOF 5600+ (AB SCIEX, USA). Detailed information on the sample preparation and analysis is available in the Supplemental Materials.

### Determination of NAD

NAD^+^ and NADH concentrations were determined using the coenzyme I NAD(H) test kit (Solarbio, China). NAD^+^ or NADH was extracted from heart tissues of mice and NRVMs, and the absorption of colorimetric solution was measured at 570 nm. The concentrations of NAD^+^ or NADH in the samples were calculated by the determination value of samples and standards.

### Mitochondrial membrane potential (MMP) measurement

JC-1 staining kit (Fudebio, China) was used for MMP analysis (An et al. 2021). Fluorescence was observed with confocal microscopy (Leica SP8, Germany). The images were analyzed with ImageJ.

### Western blotting

Proteins were extracted, quantified and analyzed as reported previously (Xiong et al. 2018; Liu et al. 2019; An et al. 2021). The primary and secondary antibodies used included anti-β-MHC, anti-ANP, anti-HO1, anti-GPX4 (1:1000, Proteintech, USA), anti-PINK1 (1:200, Santa Cruz, USA), anti-SIRT1 (1:1000, Cell Signaling Technology, USA), goat anti-rabbit and anti-mouse IgG-HRP (1:5000, Fudebio, China) antibodies.

### Quantitative real time polymerase chain reaction (qRT-PCR)

Total RNA was extracted from cardiomyocytes with TRIzol reagent (Takara, Japan) according to manufacturer’s instruction. qRT-PCR was performed on a LightCycler 480 system (Roche Diagnostics, Switzerland) as described elsewhere (An et al. 2021). The primers used are listed in Supplemental Materials (Sangon Bio, China).

### Lipid peroxidation analysis

Lipid peroxidation analysis was performed using BODIPY C11 581/591 (Invitrogen, USA) staining. Fluorescent cardiomyocytes were observed using confocal microscopy (Leica SP8, Germany) and excited by 488 and 565 nm lasers. Fluorescence at 505–550 nm (green light) and > 580 nm (red light) was detected.

### Statistical analysis

The data were expressed as the mean ± standard error of the mean (SEM). SPSS24 (IBM, USA) and GraphPad Prism 8.0 (San Diego, USA) were used for statistical analysis. Statistical differences between groups were analyzed by two-tailed t test (2 groups), or ANOVA followed by LSD or Dunnett’s T3 post hoc multiple test(more than 3 groups).

## Results

### AKG improved chronic cardiac insufficiency induced by TAC in mice

At 8 weeks after surgery, the echocardiogram showed increases in left ventricular posterior wall thickness at diastole (LVPWd), left ventricular internal dimension at systole (LVIDs), and left ventricular internal dimension at diastole (LVIDd) and decreases in left ventricular fractional shortening (LVFS) and left ventricular ejection fraction (LVEF) in TAC mice, indicating that TAC caused myocardial hypertrophy, left ventricular remodeling and cardiac insufficiency. After AKG supplementation, the LVEF and LVFS of TAC + AKG mice were increased, and the LVPWd, left ventricular posterior wall thickness at systole (LVPWs), LVIDd and LVIDs were significantly decreased (Fig. 1B–H).

### AKG improved myocardial hypertrophy and fibrosis induced by TAC in mice

**Fig. 2:**
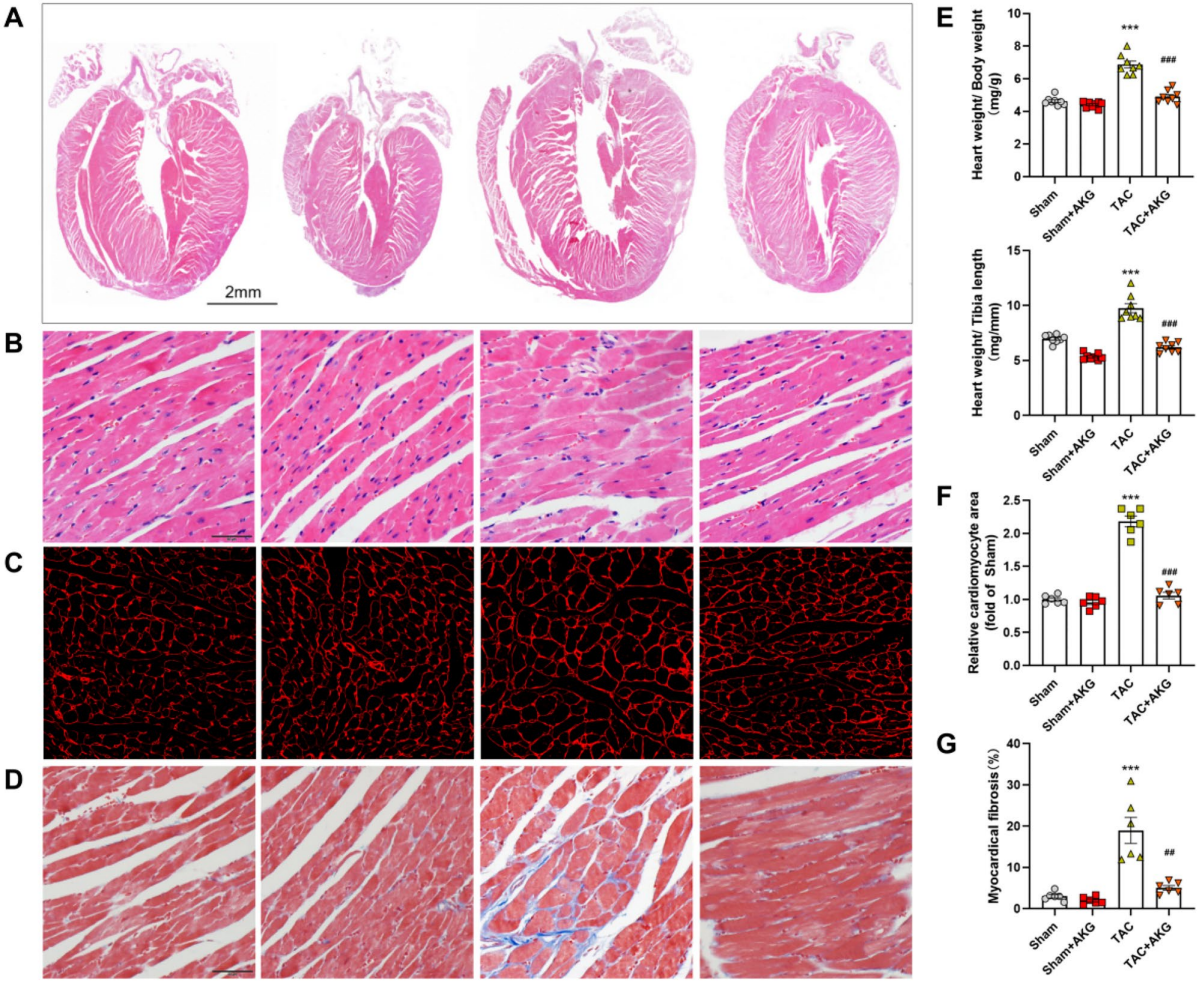
AKG alleviates myocardial hypertrophy and fibrosis in TAC mice. Whole (**A**, scale = 2 mm) and local (**B**, scale = 50 μm) heart tissue sections with HE staining. (**C**) The left ventricles tissue sections with WGA staining (scale = 50 μm). (**D**) The left ventricles tissue sections with Masson staining (scale = 50 μm). (**E**) The statistical data of HW/BW and HW/TL in each group. (**F**) Quantitative analysis of the cross sectional area of cardiomyocytes in panel C. (**G**) Quantitative analysis of myocardial fibrosis in panel D. ***P < 0.001 vs. Sham group; ^##^*P* < 0.01, ^###^*P* < 0.001 vs. TAC group

Gravimetric analysis showed that the heart weight to body weight (HW/BW) or tibia length (HW/TL) ratios of mice were increased after TAC surgery (Fig. 2E). In the HE- and WGA-stained sections, a larger cross-sectional area of cardiomyocytes were observed in left ventricles of TAC mice (Fig. 2A–C, F). Masson staining showed the more severe myocardial fibrosis of the left ventricle in TAC group (Fig. 2D, G). These effects were significantly alleviated by AKG, indicating that AKG could alleviate myocardial hypertrophy, fibrosis and cardiac insufficiency in TAC mice.

### AKG affected energy metabolites in cardiomyocytes

**Fig. 3:**
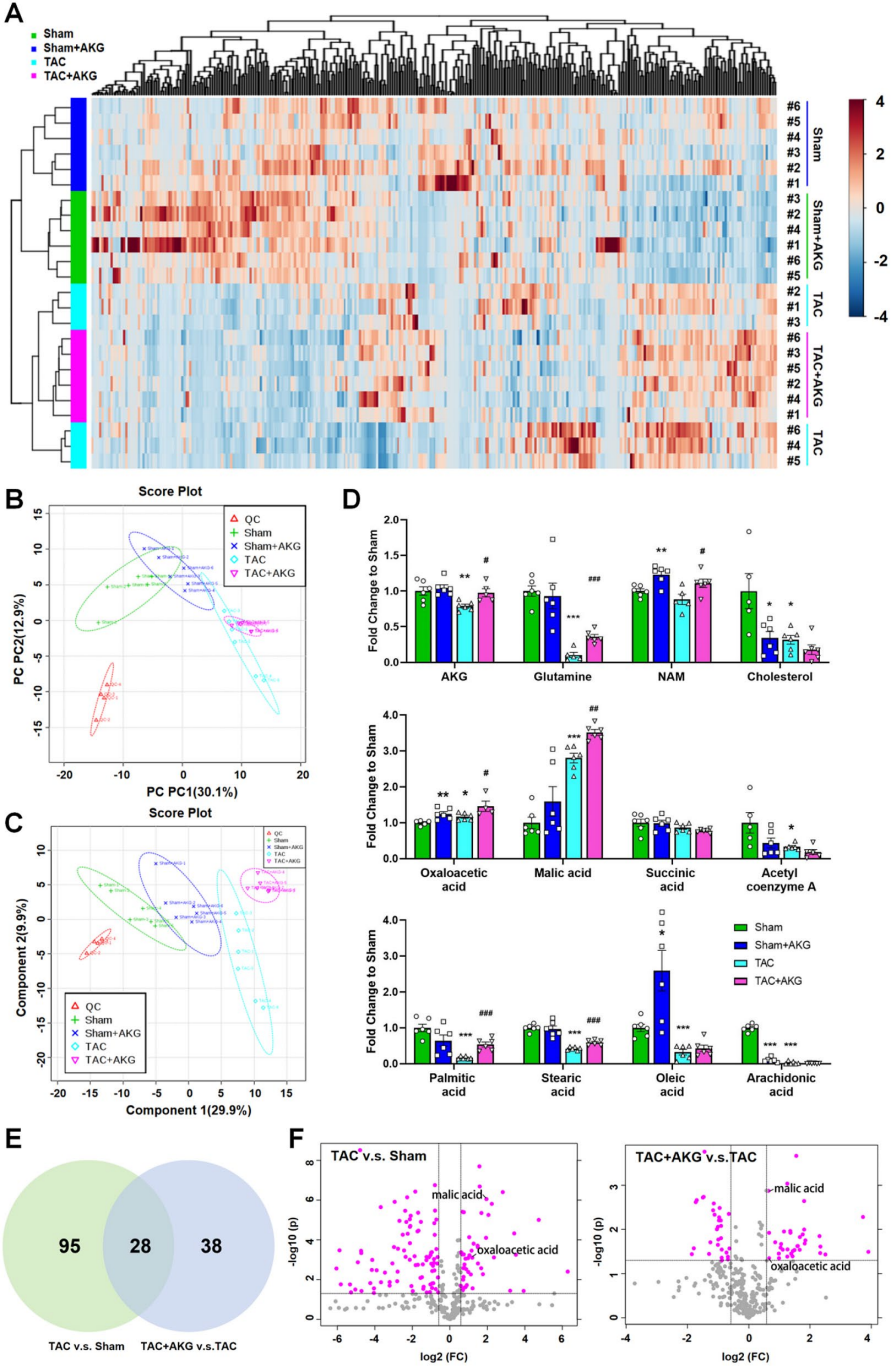
Myocardial metabolomics analysis. (**A**) Heatmap showing the total analysis of cardiac metabolites. (**B**) Principal component analysis of cardiac metabolites. (**C**) Partial least squares discriminant analysis of cardiac metabolites. (**D**) Relative quantitative analysis of α-ketoglutaric acid-related metabolites. (**E**) Venn Diagram comparing the significantly changed metabolites after TAC surgery and AKG treatment (**F**) Volcanic analysis of metabolites in the Sham group and TAC group, and in the TAC group and TAC + AKG group. *P < 0.05, **P < 0.01, ***P < 0.001 vs. Sham group; ^#^*P* < 0.05, ^###^*P* < 0.001 vs. TAC group

To explore mechanisms of the protective effect of AKG on cardiomyocytes, mouse hearts were subjected to metabolomics analysis. The LC‒MS/MS data are shown on a heatmap (Fig. 3A). The principal component analysis (PCA) plot revealed differences in Overall metabolite among the groups (Fig. 3B). We found the metabolomics characteristics of TAC mice and TAC + AKG mice overlapped in the PCA plot. However, the overlap was separated after maximizing difference between groups by the partial least squares discriminant analysis (PLS-DA) (Fig. 3C). Therefore, PLS-DA was applied to subsequent intergroup analysis.

Based on the characteristics of cardiomyocyte energy metabolism and the role of AKG in energy metabolism, we analyzed certain metabolites associated with glucose metabolism, the MAS and complementary reactions. As shown in Fig. 3D, the relative levels of AKG, glutamine, acetyl coenzyme A, succinic acid, and severe free fatty acids were decreased in the heart tissue of TAC mice. However, oxaloacetic acid and malic acid, which are involved in the malic acid shuttle, were increased after TAC surgery. As expected, levels of AKG and glutamine were restored in TAC mice. Interestingly, levels of succinic acid and acetyl-CoA were further decreased, although the levels of some free fatty acids were restored. And levels of oxaloacetic acid and malic acid were further increased after AKG treatment. The level of nicotinamide (NAM), a precursor of NAD, was also increased by AKG supplementation. Therefore, we speculated that AKG could play a role by influencing malic acid and oxaloacetic acid levels, which involved in MAS. We further compared the significant changed metabolites between sham group and TAC group, TAC group and TAC + AKG group (Supplemental Materials). As shown in Fig. 3E, twenty-eight identical metabolites changed after TAC surgery and AKG treatment, including oxaloacetic acid and malic acid (Fig. 3F). These data suggested that the MAS rather than the TCA cycle or fatty acid oxidation might be upregulated by AKG in the hearts of TAC mice.

### AKG increased the NAD^+^/NADH ratio and the expression of SIRT1 in cardiomyocytes

**Fig. 4:**
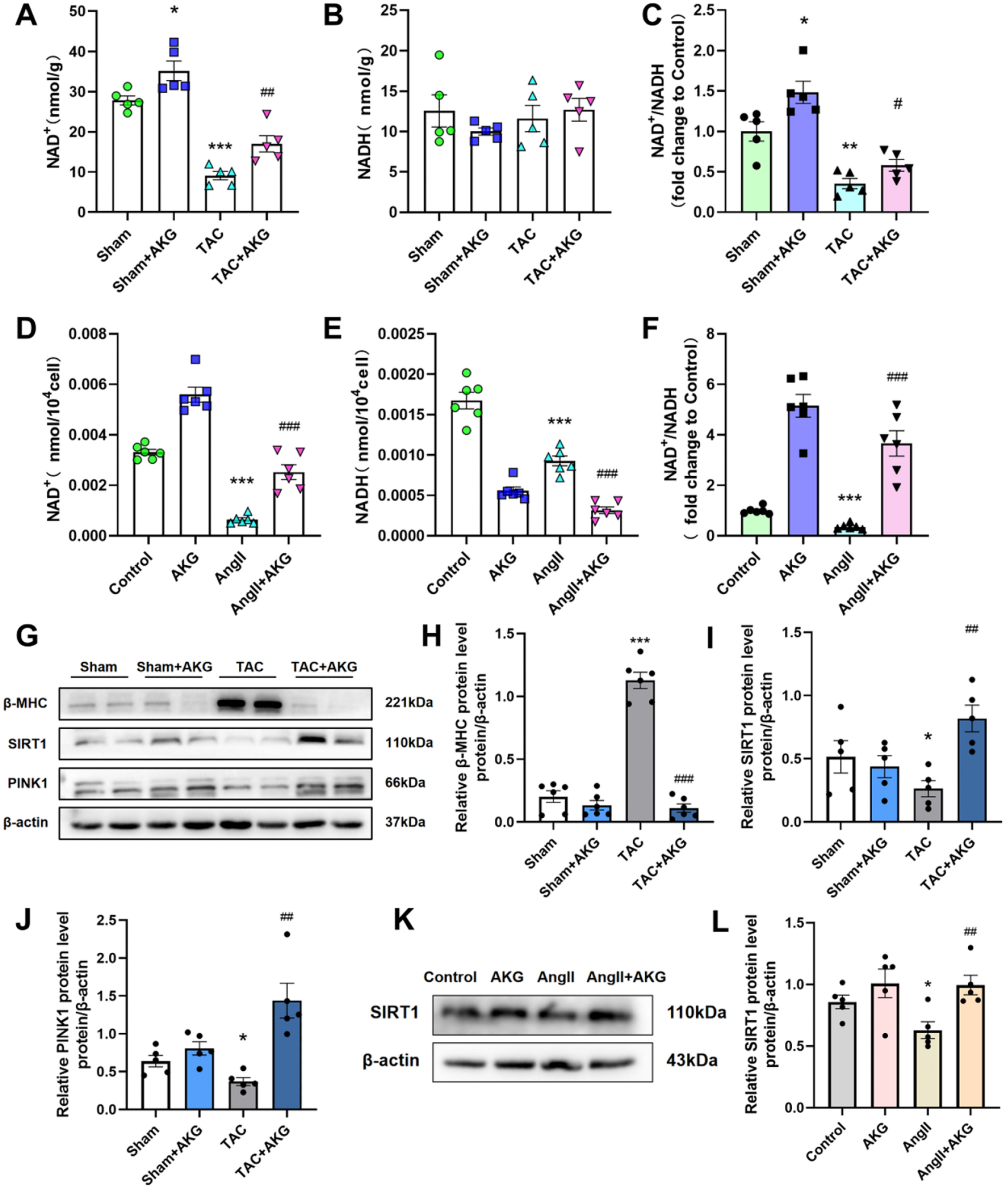
AKG upregulated NAD levels in cardiomyocytes and increased SIRT1 expression. (**A**–**C**) The level of NAD^+^, NADH, and NAD^+^/NADH radio in the hearts of mice. (**D**–**F**) The level of NAD^+^, NADH, and NAD^+^/NADH radio in vitro cardiomyocytes. (**G**) Western blot analysis of β-MHC, SIRT1, and PINK1 in the hearts of mice. (**H**–**J**) Quantitative analysis of panel G. (**K**) Western blot analysis of SIRT1 in vitro cardiomyocytes. (**L**) Quantitative analysis of panel K. *P < 0.05, **P < 0.01, ***P < 0.001 vs. Sham/Control group; ^#^*P* < 0.05, ^##^*P* < 0.01, ^###^*P* < 0.001 vs. TAC/AngII group

Based on above results, we examined levels of NAD in the myocardial tissues of mice and in vitro cardiomyocytes to verify the effects of AKG. The level of NAD^+^ and NAD^+^/NADH in heart tissues of TAC mice were significantly decreased (Fig. 4A–C), and the expression of SIRT1 was lower than in the sham group (Fig. 4G, I). After treatment with AKG, the level of NAD^+^ and the expression of SIRT1 were increased. In vitro, we observed the same changes in NAD^+^, NAD^+^/NADH (Fig. 4D–F) and SIRT1 in cardiomyocytes (Fig. 4K, L). These results indicated that AKG could upregulate NAD^+^/NADH in injured cardiomyocytes induced by TAC surgery or AngII, thus increasing the expression of SIRT1.

### AKG regulated mitophagy via the SIRT1/PINK1 pathway

**Fig. 5:**
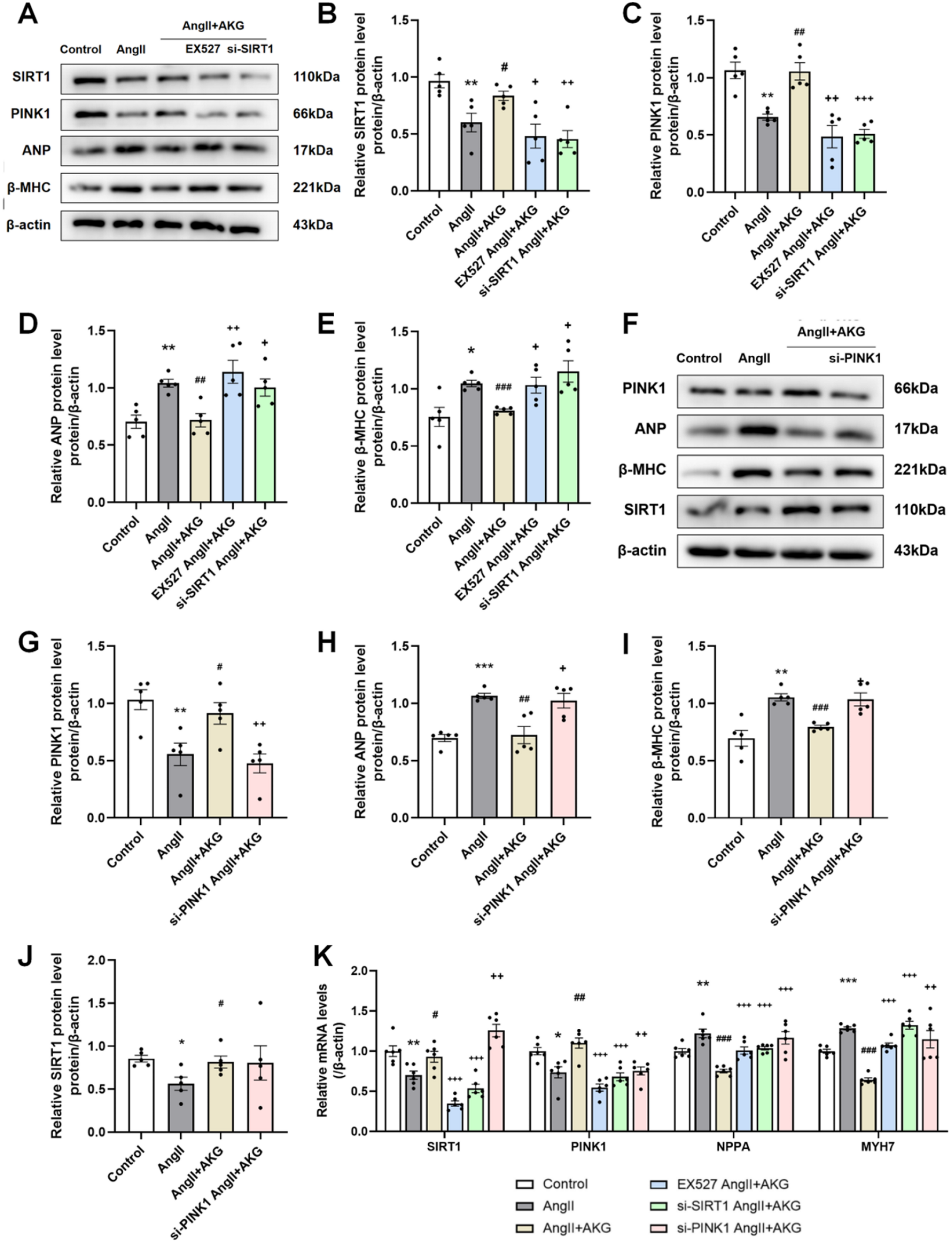
Effects of inhibiting SIRT1/PINK1 expression on myocardial hypertrophy. (**A**, **F**) Western blot analysis of protein SIRT1, PINK1, ANP and β-MHC after inhibiting SIRT1 expression (**A**) and PINK1 expression (**F**) in cardiomyocytes in vitro cardiomyocytes. (**B**–**E**) Quantitative analysis of panel A. (**G**–**J**) Quantitative analysis of panel F. (**K**) Real-time quantitative PCR analysis of SIRT1, PINK1, NPPA and MYH7 levels in cardiomyocytes. *P < 0.05, **P < 0.01, ***P < 0.001 vs. Sham/Control group; ^#^*P* < 0.05, ^##^*P* < 0.01, ^###^*P* < 0.001 vs. AngII group; ^+^*P* < 0.05, ^++^*P* < 0.01, ^+++^*P* < 0.001 vs. AngII + AKG group

**Fig. 6:**
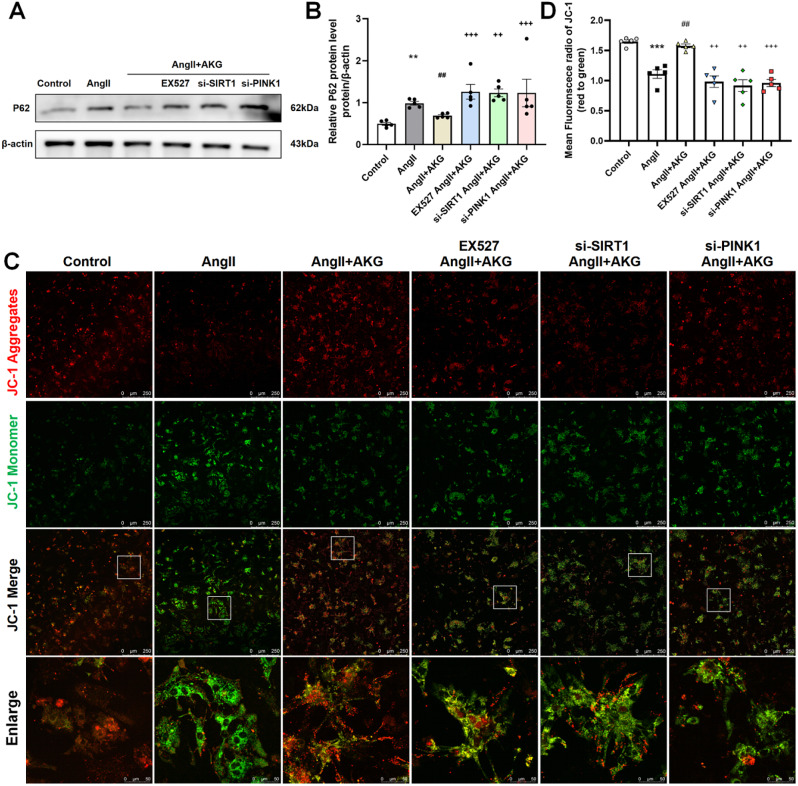
Blocking SIRT1/PINK1 abolished the AKG-mediated enhancement of mitophagy. (**A**) P62 expression in cardiomyocytes. (**B**) Quantitative analysis of panel A. (**C**) JC-1 staining fluorescence image of myocardial cells (reflecting the level of MMP, scale = 250 μm; enlarged scale = 50 μm). (**D**) Quantitative analysis of MMP, as shown by JC-1 staining (**C**). **P < 0.01, ***P < 0.001 vs. Control group; ^##^*P* < 0.01 vs. AngII group; ^++^*P* < 0.01, ^+++^*P* < 0.001 vs. AngII + AKG group

The SIRT1-PINK1 pathway can regulate autophagy (Jiang et al. 2021). In our previous studies, AKG could restore the AngII-induced decreases in PINK1 and Parkin expression and mitophagy in cardiomyocytes (Xiong et al. 2018; An et al. 2021). In addition, we found simultaneous changes in SIRT1 and PINK1 in the hearts of mice (Fig. 4G–J). To verify whether AKG regulates mitophagy through SIRT1, EX527 (a SIRT1 inhibitor) and SIRT1 siRNA were administered to AngII-induced cardiomyocytes. As shown in Fig. 5, expressions of SIRT1 and PINK1 were downregulated and β-MHC and ANP were upregulated after AngII treatment. These changes were reversed by supplementation with AKG. However, knocking down SIRT1 abolished the effects of AKG and led to a decrease in PINK1 and an increase in β-MHC and ANP compared to those in the AngII + AKG group (Fig. 5A–E). Next we evaluated whether PINK1 is a downstream target of SIRT1 by knocking down PINK1. Inhibiting PINK1 expression abolished the effects of AKG on PINK1, ANP, and MHC, but it had no significant effect on SIRT1 (Fig. 5F–J). And the same changes of above protein in transcription levels were observed by real-time qPCR (Fig. 6K).

Next, we further verified the effect of SIRT1 on mitophagy. We found that the AngII-induced increase in p62 expression (Fig. 6A, B) and decrease in MMP (Fig. 6C, D) in cardiomyocytes were significantly restored by the administration of AKG. However, when SIRT1 expression was inhibited, the effects of AKG on p62 protein expression and MMP were reduced, which was similar to when PINK1 expression was inhibited (Fig. 6). These results confirm that AKG reduces cardiomyocyte injury induced by AngII by promoting mitophagy through SIRT1-PINK1 pathway.

### AKG attenuated AngII-induced ferroptosis in cardiomyocytes

**Fig. 7:**
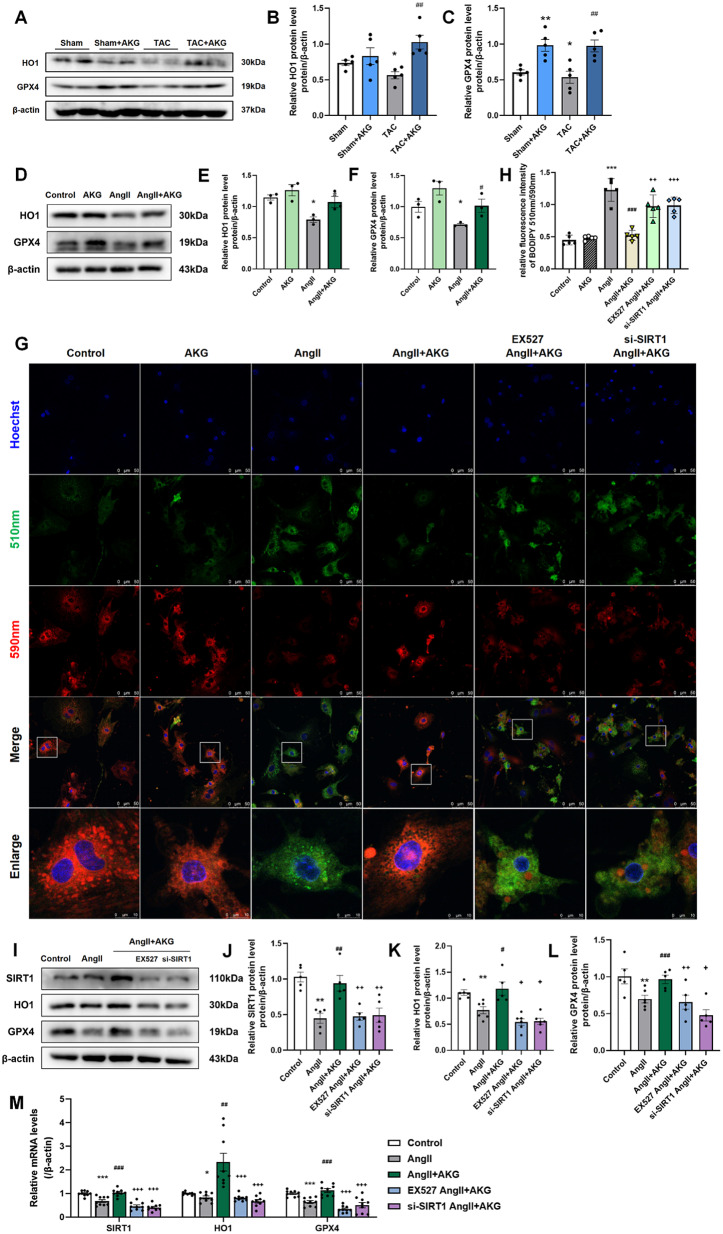
AKG inhibits Ang II-induced ferroptosis in cardiomyocytes via SIRT1. (**A**, **D**) Western blot analysis of HO1 and GPX4 in the hearts of mice (**A**) and vitro cardiomyocytes (**D**). (**B**, **C**) Quantitative analysis of panel A. (**E**, **F**) Quantitative analysis of panel D. (**G**) BODIPY C11 581/591 fluorescence staining of cardiomyocytes (scale = 50 μm, enlarged scale = 10 μm). (**H**) Quantitative analysis of panel G. (**I**) Western blot analysis of SIRT1, HO1 and GPX4 protein expression after inhibiting SIRT1 expression in vitro cardiomyocytes. (**J**–**L**) Quantitative analysis of panel I. (**M**) Relative quantitative analysis of SIRT1, HO1 and GPX4 transcription levels in cardiomyocytes, as detected by real-time quantitative PCR. **P < 0.05, **P < 0.01, ***P < 0.001 vs. Sham/Control group; ^#^*P* < 0.05, ^##^*P* < 0.01, ^###^*P* < 0.001 vs. TAC/AngII group; ^+^*P* < 0.05, ^++^*P* < 0.01, ^+++^*P* < 0.001 vs. AngII + AKG group

Ferroptosis is regulated by SIRT1 pathway and autophagy (Ma et al. 2020; Liu et al. 2023). Next, we further verified the effect of AKG on ferroptosis. Figure 7A–C shows the decreased expressions of HO1 and GPX4 in hearts of TAC mice. And the same changes were observed in cardiomyocytes induced by AngII in vitro (Fig. 7D–F). In addition, AngII significantly increased lipid peroxidation in cardiomyocytes (Fig. 7G, H). However, AKG treatment reversed these changes, which indicated that AKG could reduce TAC surgery- or AngII-induced ferroptosis in cardiomyocytes (Fig. 7A–G).

To verify whether SIRT1 is involved in the anti-ferroptotic effect of AKG, we knocked down SIRT1 in cardiomyocytes before stimulation with AngII or AKG in vitro. The inhibiting SIRT1 expression attenuated the effect of AKG on reducing lipid peroxidation in AngII-induced cardiomyocytes (Fig. 7G, H). And the expression of HO1 and GPX4 were also decreased when SIRT1 was inhibited compared with those in the AngII + AKG group (Fig. 7I–L). The same changes in transcription levels were observed by real-time qPCR (Fig. 7M). In conclusion, AKG inhibited ferroptosis in cardiomyocytes by activating SIRT1-HO1/GPX4, and inhibiting SIRT1 expression weakened the anti-ferroptotic effect of AKG.

## Discussion

In this study, we investigated the underlying mechanism of AKG-mediated amelioration of chronic cardiac insufficiency induced by stress overload in mice. We found AKG could affect cardiometabolic changes in TAC mice, especially increasing intracellular NAD^+^ levels. The increase in NAD^+^ activated the expression of SIRT1 and downstream proteins, including PINK1 and GPX4, promoted mitophagy and inhibited ferroptosis in cardiomyocytes, thereby reducing damage to cardiomyocytes and improving cardiac systolic function. When SIRT1 was knocked down, the protective effect of AKG was inhibited. These results indicate the mechanism by which AKG protects cardiomyocytes.

Energy metabolism is altered in the failing cardiomyocytes, especially mitochondrial oxidative phosphorylation, and the levels of products of the TCA cycle are also changed (van Bilsen et al. 2004; Peng et al. 2019; Lopaschuk et al. 2021). AKG is a metabolite of the TCA cycle, and AKG supplementation could improve cardiac dysfunction in TAC mice (Asadi Shahmirzadi et al. 2020; An et al. 2021). We surmised that this could be attributed to the improvement of the TCA cycle. Unexpectedly, the decreased levels of succinic acid and acetyl-CoA could not support this hypothesis in our study. In the meantime, the levels of malic acid and oxaloacetic acid were increased in both the TAC group and TAC + AKG group, suggesting that AKG upregulates metabolite concentrations through other pathways. The MAS is a key participant in cytoplasmic and mitochondrial NAD redox (Houtkooper et al. 2010). Through the conversion of oxaloacetic acid and malic acid, NADH can be oxidized to NAD+, which enters mitochondria and participates in the energy metabolism (Lee et al. 2016). In the early stage of HF, the increased MAS flux contributes to the production of ATP by aerobic glycolysis to provide energy for the myocardium (Allard et al. 1994; Nascimben et al. 2004; Lewandowski et al. 2007). This may result in increases and cumulative levels of malic acid and oxaloacetic acid. With further aggravation of HF, decreases in intermediate substances and the high acetylation of MAS proteins decrease the activity of MAS and the ratio of NAD^+^/NADH (Lee et al. 2016).

The high NAD^+^/NADH ratio is necessary to maintain energy metabolic processes and mitochondrial function (Imai and Guarente 2014; Lee et al. 2016; Chiao et al. 2021). In addition, NAD^+^/NADH affects the expression of SIRTs and thus regulates protein acetylation (Karamanlidis et al. 2013; Imai and Guarente 2014; Chiao et al. 2021). Reportedly, supplementation with NAD precursor substances could enlarge the NAD pool, and activating MAS could increase the internal circulation of NAD, which regulates NAD^+^/NADH (Rupert et al. 2000; Lee et al. 2016). In our study, significant increases in malic acid, oxaloacetic acid and niacinamide levels in the heart were observed in the AKG + TAC group. And supplementation with AKG increased the level of NAD^+^ and expression of SIRT1 in cardiomyocytes in vivo or vitro.

Previous studies suggest that autophagy and ferroptosis are regulated by different signaling pathways and are involved in cardiovascular diseases (Li et al. 2021; Ma et al. 2022; Packer 2023). However, it has recently been found that ferroptosis is associated with autophagy (Zhou et al. 2020; Yang et al. 2023). In ischemia/reperfusion (I/R) injury after kidney transplantation, mitophagy occurs simultaneously with ferroptosis and is coregulated by antioxidants (Granata et al. 2022). In our study, AKG supplementation can regulate SIRT1 and simultaneously affect mitophagy- and ferroptosis-related pathways, thereby protecting cardiomyocytes. Notably, excessive autophagy promotes ferroptosis in cell injury, which is essentially caused by the activation of oxidative stress pathways (Chen et al. 2021, 2022; Jankauskas et al. 2023). When mitophagy is inhibited in HF, the cascade amplification reaction of mitochondrial damage will also lead to oxidative stress in cardiomyocytes (Xiong et al. 2018; An et al. 2021). Therefore, appropriate upregulation of mitophagy could also inhibit ferroptosis in cardiomyocytes in HF.

Another change in the energy metabolism of cardiomyocytes after HF is a decrease in fatty acid metabolism. This is a manifestation of cardiomyocyte damage and a self-protective response, which maintains myocardial energy requirements by reducing fatty acid metabolism, preventing large oxygen consumption, and increasing glucose or ketone body metabolism (Honka et al. 2021). Interestingly, AKG supplementation increased the intake of fatty acids by cardiomyocytes but reduced the production of acetyl-CoA, a fatty acid oxidation product. This suggests that fatty acid oxidation might be inhibited by AKG treatment to further strengthen hypoxic energy metabolism and save myocardial energy after myocardial injury. Notably, the increase in fatty acids could also aggravate the damage to cardiomyocytes (Pei et al. 2021). Polyunsaturated fatty acids, especially arachidonic acid, are substrates of lipid peroxidation and are metabolized through the ACSL4 pathway to promote ferroptosis (Liao et al. 2022; Kong et al. 2023; Yui et al. 2023). In our study, fatty acid uptake by cardiomyocytes was selective, and arachidonic acid levels were further reduced after AKG supplementation, which could be the reason for the reduction in lipid peroxidation in cardiomyocytes. We also found that AKG supplementation increased glutamine levels in cardiomyocytes, which could affect the expression of GPX4 (Bott et al. 2019; Watanabe et al. 2021). In conclusion, the regulatory effect of AKG on ferroptosis may involve multiple pathways. However, a previous study illustrated that AKG could promote ferroptosis in tumor cells of diffuse large B-cell lymphoma (Cai et al. 2023). This discrepancy with our results may be due to changes in tumor cells and differences between cell species.

In addition, we found that HO1, a cell protective, anti-inflammatory and antioxidant enzyme, plays a controversial role in ferroptosis (Gottlieb et al. 2012). Studies have shown that bilirubin or biliverdin and carbon monoxide, which are metabolites of HO1, can reduce oxidative stress and inhibit ferroptosis (Ryter et al. 2006; Sugimoto et al. 2012). However, ferrous ions produced during HO1 metabolism can promote ferroptosis (Papanikolaou and Pantopoulos 2005; Shi et al. 2023). In our study, the change in HO1 expression was synchronized with SIRT1 and GPX4, and ferroptosis was inhibited. These findings are consistent with the results of other studies on HO1 activation through SIRT1 (Li et al. 2021; Dang et al. 2022). Therefore, some scholars have suggested the effect of HO1 on ferroptosis depends on the intensity of protein expression. Moderate activation of HO1 can play a protective role by clearing ROS. Overactivation of HO1 increases unstable ferrous ions, leading to ROS overload and ferroptosis (Chiang et al. 2018).

## Conclusion

In this study, we demonstrate that AKG promotes mitophagy, inhibits ferroptosis in cardiomyocytes and alleviates myocardial cell damage through the NAD^+^-SIRT1 pathway. These changes improved myocardial hypertrophy, fibrosis, and chronic cardiac insufficiency in mouse model of HF induced by TAC. These results indicate that AKG has certain potential in the treatment of HF. However, there are some limitations in our study. First, we could only predict the increase in MAS flux through the increase in several metabolites of MAS. The source of NAD + and the role of MAS after AKG supplementation remain to be explained by more studies. Second, there are many metabolites affected by AKG as a metabolic intermediate, and whether there are other mechanisms involved in cardiomyocyte protection remains to be further explored.

### Electronic supplementary material

Below is the link to the electronic supplementary material.

**Supplementary Material 1:** 1. Supplemental Methods include Section 2.5. Untargeted Metabolomics and Section 2.9. The primers of qRT-PCR; 2. **Supplemental Table 1:** Cardiac difference metabolites between Sham group and TAC group; 3. **Supplemental Table 2:** Cardiac difference metabolites between TAC group and TAC+AKG group

## Data Availability

The datasets used and/or analysed during the current study are available from the corresponding author on reasonable request.
