# Supplementary material for: α-Ketoglutarate improves cardiac insufficiency through NAD+-SIRT1 signaling-mediated mitophagy and ferroptosis in pressure overload-induced mice
Source: Mol Med. 2024 Jan 22;30:15. doi: 10.1186/s10020-024-00783-1 (PMC10804789; doi:10.1186/s10020-024-00783-1)
Supplement: Supplementary file 1 — Supplementary Material 1: 1. Supplemental Methods include Section 2.5. Untargeted Metabolomics and Section 2.9. The primers of qRT-PCR; 2. Supplemental Table 1: Cardiac difference metabolites between Sham group and TAC group; 3. Supplemental Table 2: Cardiac difference metabolites between TAC group and TAC+AKG group [file 10020_2024_783_MOESM1_ESM.docx]

**Supplemental Materials**

**α-Ketoglutarate improves cardiac insufficiency through NAD^+^-SIRT1** **signaling-mediated mitophagy and ferroptosis in pressure overload-induced mice**

Hao Yu, Daojing Gan, Zhen Luo, Qilin Yang, Dongqi An, Hao Zhang, Yingchun Hu, Zhuang Ma, Qingchun Zeng, Dingli Xu, Hao Ren

**Page 2: Supplemental Methods**

**Page 4: Supplemental Table 1**

**Page 9: Supplemental Table 2**

**Supplemental Methods**

**2.5. Untargeted Metabolomics**

Heart samples were snap-frozen and stored at −80 ℃. Fifty milligrams of tissue was taken from each myocardium sample and then extracted in 500 μL of methanol. After rotating for 1 h, the sample tubes were centrifuged at 15000 rpm and −4°C for 10 min, and then the supernatant was transferred to a new tube. The supernatant of each group was 10 μl mixed in a tube as the mixing sample.

Liquid chromatography‒mass spectrometry (LC‒MS)-based analyses were performed on a Sepax GP-C18 Column (1.8 µm 120 Å 2.1 mm*150 mm) coupled to a Thermo Ultimate 3000 (Thermo Fisher Scientific, USA). The chromatographic gradient was run at a flow rate of 0.3 mL/min as follows: 0–10 min: linear gradient from 5% to 70% Buffer B (CH_3_CN, mixed with 5% to 70% of Buffer A: 0.1% CH_2_O_2_); 10–17 min: linear gradient from 70% to 100% Buffer B; 17–18 min: hold at 100% Buffer B; 17–19 min: linear gradient from 100% to 5% Buffer B; 19–21 min: hold at 5% Buffer B. The positive and negative ion mass detected was by electrospray ionization (ESI) by a TripleTOF 5600+ (AB SCIEX, USA). The ESI source conditions were as follows: ion source gas 1 (gas 1): 50; ion source gas 2 (gas 2): 50; curtain gas (CUR): 25; source temperature: 500℃ (positive ion) and 450℃ (negative ion); ion spray voltage floating (ISVF) 5500 V (positive ion) and 4400 V (negative ion); TOF MS scan range: 100-1200 Da; product ion scan range: 50-1000 Da; TOF MS scan accumulation time 0.2 s; and product ion scan accumulation time 0.01 s. Secondary mass spectra were obtained using information-dependent acquisition (IDA) and declustering potential (DP) using a high sensitivity model: ±60 V, collision energy: 35±15 eV.

The LC‒MS data were preprocessed using Analysis Base File Converter Software and MS-DIAL 4.70X Software. The extracted peak information was compared with the full MassBank, Respect and GNPS databases.

**2.9. Quantitative real time polymerase chain reaction (qRT-PCR)**

**The primers of qRT-PCR**

| Genus | mRNA | Forward | Reverse |
| --- | --- | --- | --- |
| rat | β-actin | CTGAGAGGGAAATCGTGCGTGAC | AGGAAGAGGATGCGGCAGTGG |
| rat | Gpx4 | CCAGCAACAGCCACGAGTTCC | CACACGCAACCCCTGTACTTATCC |
| rat | Hmox1 | GGGTCAGGTGTCCAGGGAAGG | TGGGTTCTGCTTGTTTCGCTCTATC |
| rat | MYH7 | CCAGAACACCAGCCTCATCAACC | CACCGCCTCCTCCACCTCTG |
| rat | Nppa | GAGCGAGCAGACCGATGAAGC | TCCATCTCTCTGAGACGGGTTGAC |
| rat | PINK1 | GAAGCCACCATGCCCACACTG | CTGCTCCCTTTGAGACGACATCTG |
| rat | SIRT1 | ACGCCTTATCCTCTAGTTCCTGTGG | CGGTCTGTCAGCATCATCTTCCAAG |

**Supplemental Table 1:** Cardiac difference metabolites between Sham group and TAC group

| **Compounds** | **p.value** |
| --- | --- |
| Arachidonic Acid | 3.15E-09 |
| sn-Glycero-3-phosphocholine | 2.03E-08 |
| Tetrahydrogambogic Acid | 1.88E-07 |
| 1-methyl-2-undecylquinolin-4-one | 2.07E-07 |
| N-(4-((6aR,8aS)-4-hydroxy-6a,8a,9-trimethyl-3,4,5,6,6a,6b,7,8,8a,8b,11a,12,12a,12b-tetradecahydro-1H-naphtho[2',1':4,5]indeno[2,1-b]furan-10-yl)-2-methylbutyl)acetamide | 3.77E-07 |
| (S)-2-((1S,4S)-4-(((S)-2-((tert-butoxycarbonyl)amino)-3-phenylpropanamido)methyl)cyclohexanecarboxamido)-3-phenylpropanoic acid | 4.02E-07 |
| Phosphatidylethanolamine lyso alkenyl | 7.31E-07 |
| N-(3-Methoxybenzyl)(9Z,12Z,15Z)-octadeca-9,12,15-trienamide | 1.24E-06 |
| methyl 4-((10R,13R)-3-hydroxy-10,13-dimethyl-2,3,4,7,8,9,10,11,12,13,14,15,16,17-tetradecahydro-1H-cyclopenta[a]phenanthren-17-yl)pentanoate | 1.56E-06 |
| CHOLINE | 2.29E-06 |
| Stearic acid | 3.40E-06 |
| (3R,7R,8R,8aS)-3,4'-dihydroxy-7'-(2-hydroxyethyl)-4,4,7,8a-tetramethylspiro[2,3,4a,5,6,7-hexahydro-1H-naphthalene-8,2'-3,8-dihydrofuro[2,3-e]isoindole]-6'-one | 3.51E-06 |
| PFAP-PAP | 3.87E-06 |
| 4,7-dimethyl-7-(4-methylpent-3-enyl)bicyclo[2.2.1]heptan-3-ol | 4.02E-06 |
| allantoin | 4.38E-06 |
| Dodemorph | 4.45E-06 |
| Phosphocholine | 5.60E-06 |
| L-methionine sulfone | 6.08E-06 |
| Conivaptan HCl (Vaprisol) | 9.16E-06 |
| (4S,4aR,8aS)-4-[(3R)-3-hydroxy-3-methylpent-4-enyl]-3,4a,8,8-tetramethyl-5,6,7,8a-tetrahydro-4H-naphthalen-1-one | 9.69E-06 |
| Hecogenin | 1.00E-05 |
| 2-amino-5-[2-[[2,3-dihydroxy-2-(1-hydroxyethyl)butanoyl]oxymethyl]-4-hydroxyanilino]-5-oxopentanoic acid | 1.13E-05 |
| 2-methyl-4-(pyridin-2-yl)but-3-yn-2-ol | 1.27E-05 |
| Glutamine | 1.28E-05 |
| 1,2-dihydroxyheptadec-16-en-4-yl acetate | 1.48E-05 |
| Flavone base + 4O, C-Hex-FeruloylHex | 2.01E-05 |
| Palmitic acid | 2.23E-05 |
| methyl 4-(4,6-dihydroxy-5-methoxy-2,5-dimethyl-3-oxocyclohexen-1-yl)oxy  -2-hydroxy-3,6-dimethylbenzoate | 3.49E-05 |
| Secopenitrem D_120253 | 3.77E-05 |
| ISOPALMITIC ACID | 4.77E-05 |
| [3,4,5-trihydroxy-6-(3,4,5-trihydroxybenzoyl)oxyoxan-2-yl]methyl 3,4,5-trihydroxybenzoate | 4.95E-05 |
| forskolin | 5.51E-05 |
| LPC 18:1 | 5.70E-05 |
| d-LIMONENE | 7.39E-05 |
| 9-hydroxy-5b,8,11a-trimethyl-1-prop-1-en-2-yl-1,2,3,4,5,6,7,7a,9,10,11,11b,12,13,13a,13b-hexadecahydrocyclopenta[a]chrysene-3a,5a,8-tricarboxylic acid | 7.95E-05 |
| Ophiopogonoside A | 1.48E-04 |
| Prednisolone | 1.55E-04 |
| Rhodojaponin II | 1.97E-04 |
| Colladine [M-H2O+H]+ | 2.33E-04 |
| 2-hydroxy-1-isopentyl-4-methyl-1H-imidazol-5(4H)-one | 2.34E-04 |
| (2R,4aR,7S,8S,8aR)-8-[(Z)-5-hydroxy-3-methylpent-3-enyl]-4,4,7,8a-tetramethyl-2,3,4a,5,6,8-hexahydro-1H-naphthalene-2,7-diol | 2.82E-04 |
| haplamine | 2.94E-04 |
| (4aR,5S)-9,9a-dihydroxy-3,4a,5-trimethyl-5,6,7,8,8a,9-hexahydro-4H-benzo[f][1]benzofuran-2-one | 3.39E-04 |
| Serine-Cholic Acid | 3.43E-04 |
| ethyl 4-(1-(3,4-diethoxyphenyl)-6,7-diethoxy-3-oxo-3,4-dihydroisoquinolin  -2(1H)-yl)benzoate | 3.48E-04 |
| Isomajdine | 3.61E-04 |
| 2-[(4S,5S,5aS,9aS)-4-methoxy-6,6,9a-trimethyl-5-[(2E,4E,6E)-octa-2,4,6-trienoyl]oxy-1-oxo-4,5,5a,7,8,9-hexahydro-3H-benzo[e]isoindol-2-yl]pentanedioic acid | 4.22E-04 |
| 6-Hydroxycaproic acid | 4.24E-04 |
| Tobramycin 1-N- or 3-N-carbamoyl | 4.40E-04 |
| 3-Phosphonopropionic acid | 4.60E-04 |
| 2-methoxy-4-pentadecylbenzoic acid | 5.33E-04 |
| potassium (1R,5aR)-5a,5b,8,8,11a-pentamethyl-9-oxo-1-(prop-1-en-2  -yl)icosahydro-1H-cyclopenta[a]chrysene-3a-carboxylate | 5.37E-04 |
| C16 Lactosyl Ceramide (d18:1/16:0) | 5.63E-04 |
| Famotidine | 5.95E-04 |
| valeramide | 6.00E-04 |
| ugaferin | 6.75E-04 |
| ginnalin A | 7.20E-04 |
| Veratrosine | 7.35E-04 |
| S-((3S,10R,13R)-10,13-dimethyl-17-octyl-2,3,4,7,8,9,10,11,12,13,14,15,16,17-tetradecahydro-1H-cyclopenta[a]phenanthren-3-yl) O-(4-nitrophenyl) carbonothioate | 7.48E-04 |
| Antimycin A2 | 7.53E-04 |
| Phosphatidylethanolamine alkenyl | 7.67E-04 |
| 4-hydroxy-3-[(4-hydroxy-6-methyl-2-oxopyran-3-yl)methyl]-6-methylpyran-2-one | 7.89E-04 |
| 2-(2-hydroxybut-3-en-2-yl)-3a,6,6,9a-tetramethyl-2,4,5,5a,7,8,9,9b-octahydro-1H-benzo[e][1]benzofuran-4,5-diol | 8.30E-04 |
| 2-methylidene-4-[(2R,3R,4S,5S,6R)-3,4,5-trihydroxy-6-(hydroxymethyl)oxan-2-yl]oxybutanoic acid | 8.82E-04 |
| Phosphatidylcholine lyso | 9.42E-04 |
| Vindoline | 1.01E-03 |
| N-(3-(dimethylamino)propyl)-2-((3,4,8,8-tetramethyl-2-oxo-2,8,9,10-tetrahydropyrano[2,3-f]chromen-5-yl)oxy)acetamide | 1.03E-03 |
| 6-Hydroxysumatrol | 1.07E-03 |
| Linoleyl Carnitine | 1.35E-03 |
| 8-benzoyl-1,5,5-trimethyl-6,15-dioxatetracyclo[9.3.1.0,.0,]pentadeca-7(12),8,10-trien-9-ol | 1.47E-03 |
| Dehydropachymic acid | 1.55E-03 |
| (Z)-2-((Z)-1-(hydroxyimino)ethyl)-6,6-dimethylbicyclo[3.1.0]hexan-3-one oxime | 1.55E-03 |
| hydroquinidine | 1.58E-03 |
| securinine | 1.61E-03 |
| C18(Plasm)-18:1 PC | 1.72E-03 |
| Roquefortine C | 1.73E-03 |
| apiosylskimmin | 1.81E-03 |
| 2-Thiouridine | 1.88E-03 |
| 2-hydroxy-4-methoxy-3,5-bis(3-methylbut-2-enyl)-6-(2-phenylethyl)benzoic acid | 2.04E-03 |
| (R)-4-aminoisoxazolidin-3-one | 2.57E-03 |
| [(2R,3R,4S,5S,6R)-2-(acetyloxymethyl)-3,5-dihydroxy-6-[(2S,3R)-2,3,4-trihydroxybutoxy]oxan-4-yl] hexanoate | 2.65E-03 |
| Isovitexin | 2.67E-03 |
| Bullatine G | 2.79E-03 |
| LPC 16:0 | 2.98E-03 |
| sodium (2R)-2-((2R,5S,6R)-6-((3E,5E)-6-((3aR,4S,7aS)-4-(1H-pyrrole-2  -carbonyl)-2,3,3a,4,5,7a-hexahydro-1H-inden-5-yl)hexa-3,5-dien-3-yl)-5-methyltetrahydro-2H-pyran-2-yl)butanoate | 3.29E-03 |
| LPE 16:0 | 3.41E-03 |
| Mesaconine | 3.52E-03 |
| Gentiannine | 3.75E-03 |
| 1-palmitoyl-2-oleoyl-sn-glycero-3-phosphocholine | 4.00E-03 |
| (1S,2S,6S,9S,10S,11R,12R,13S,14S,15S,16R,18S,19S,22S,23R,25R)-6,10,19-trimethyl-24-oxa-4-azaheptacyclo[12.12.0.0,.0,.0,.0,.0,]hexacosane-10,12,13,14,16,22,23-heptol | 4.02E-03 |
| Methyl gallate | 4.12E-03 |
| N-Fructosyl S-(2-carboxypropyl)glutathione | 4.46E-03 |
| Indinavir | 5.32E-03 |
| (E)-1-[3-[(2,3-dihydroxyphenyl)methyl]-2,4-dihydroxy-6-methoxyphenyl]-3-phenylprop-2-en-1-one | 5.48E-03 |
| Antimycin A4 | 7.26E-03 |
| (2R,3S,4S,5R,6R)-2-[[(2R,3R,4R)-3,4-dihydroxy-4-(hydroxymethyl)oxolan-2-yl]oxymethyl]-6-[(2E)-3,7-dimethylocta-2,6-dienoxy]oxane-3,4,5-triol | 7.67E-03 |
| (3S,6S,6aR,7R,7aR,8S,9R,10S,11aR,12R,13S,14R)-1-ethyl-3-(hydroxymethyl)-6,8,10,13-tetramethoxytetradecahydro-1H-3,6a,12-(epiethane[1,1,2]triyl)-7,9-methanonaphtho[2,3-b]azocine-11a,12-diol | 7.95E-03 |
| 2-hydroxy-4-methoxy-3,5-bis(3-methylbut-2-enyl)-6-pentylbenzoic acid | 8.00E-03 |
| (3E,5S,7R,8R,11E,13S,15R,16R)-3,5,7,11,13,15-hexamethyl-8,16-bis(1,3-oxazol-5-ylmethyl)-1,9-dioxacyclohexadeca-3,11-diene-2,10-dione | 8.29E-03 |
| Canarione | 1.01E-02 |
| 3,4-dimethoxy-myricetin-3-O-dideoxyhexosyl(1-2)-dideoxyhexoside | 1.21E-02 |
| 6-acetyloxy-7-ethenyl-1,4a,7-trimethyl-3,4,4b,5,6,8,10,10a-octahydro-2H-phenanthrene-1-carboxylic acid | 1.34E-02 |
| (3S,3'R,3'aS,6'S,6aS,6bS,7'aR,9R,11bR)-3-hydroxy-3',6',10,11b-tetramethylspiro[1,2,3,4,6,6a,6b,7,8,11a-decahydrobenzo[a]fluorene-9,2'-3a,4,5,6,7,7a-hexahydro-3H-furo[3,2-b]pyridine]-11-one | 1.37E-02 |
| Sorbitane Monooleate - Polysorbate 20 in-source fragment | 1.51E-02 |
| 4-amino-2-hydroxy-5-[[1-hydroxy-1-(5-oxo-6-bicyclo[4.1.0]hept-3-enyl)propan-2-yl]amino]-5-oxopentanoic acid | 1.54E-02 |
| Solidagenone | 1.58E-02 |
| DIETHANOLAMINE | 1.74E-02 |
| 5-[1,2,4a-trimethyl-5-(3-methylbutanoyloxymethyl)-2,3,4,7,8,8a-hexahydronaphthalen-1-yl]-3-methylpentanoic acid | 1.76E-02 |
| methyl (1S,4S,8R,11S,14S)-11-ethyl-12-oxo-7,9,13-trioxatetracyclo[6.5.1.0,.0,]tetradeca-2,5-diene-5-carboxylate | 1.77E-02 |
| Goniothalenol | 1.88E-02 |
| PFAP-FT_diPAP | 2.01E-02 |
| Scytophycin E | 2.01E-02 |
| [(2R,3S,4S,5R,6S)-6-[(2S,3R,4S,5R,6R)-6-[[(1S,3R,4S,4aR,8aR)-4-[(3S)-3-[(2S,3R,4R,5R,6R)-3,4-dihydroxy-6-methyl-5-[(2S,3R,4R,5R,6S)-3,4,5-trihydroxy-6-methyloxan-2-yl]oxyoxan-2-yl]oxy-3-methylpent-4-enyl]-3,4,8,8a-tetramethyl-1,2,3,4a,5,6-hexahydronaphthalen-1-yl]oxy]-4,5-dihydroxy-2-methyloxan-3-yl]oxy-3,4,5-trihydroxyoxan-2-yl]methyl acetate | 2.13E-02 |
| Phosphatidylethanolamine | 2.31E-02 |
| PYRIDOXAMINE | 2.36E-02 |
| inosine | 2.64E-02 |
| (3a1R,4R,5S,5aR,10bR)-methyl 4-acetoxy-3a-ethyl-5-hydroxy-8-methoxy-6-methyl-3a,3a1,4,5,5a,6,11,12-octahydro-1H-indolizino[8,1-cd]carbazole-5-carboxylate | 2.66E-02 |
| 8-[4,5-dihydroxy-6-(hydroxymethyl)-3-[3,4,5-trihydroxy-6-(hydroxymethyl)oxan-2-yl]oxyoxan-2-yl]-5,7-dihydroxy-2-(4-hydroxyphenyl)chromen-4-one | 2.74E-02 |
| Strophanthidin | 2.78E-02 |
| Lacidipine | 2.93E-02 |
| 4-Hydroxybenzylcyanide | 3.04E-02 |

**Supplement Table 2:** Cardiac difference metabolites between TAC group and TAC+AKG group

| **Compounds** | **p.value** |
| --- | --- |
| Rhodojaponin II | 1.73E-04 |
| Palmitic acid | 2.14E-04 |
| Hypoxanthine | 9.14E-04 |
| Malic acid | 1.06E-03 |
| BIOTIN | 1.33E-03 |
| 2-[(4S,5S,5aS,9aS)-4-methoxy-6,6,9a-trimethyl-5-[(2E,4E,6E)-octa-2,4,6-trienoyl]oxy-1-oxo-4,5,5a,7,8,9-hexahydro-3H-benzo[e]isoindol-2-yl]pentanedioic acid | 1.83E-03 |
| Deacetylvindoline | 1.89E-03 |
| Dihydrosphingosine | 2.17E-03 |
| Isomajdine | 2.26E-03 |
| Indinavir | 2.40E-03 |
| PFAP-diPAP | 2.57E-03 |
| Mesaconine | 3.26E-03 |
| Schisandrin A | 3.67E-03 |
| N-(3-(dimethylamino)propyl)-2-((3,4,8,8-tetramethyl-2-oxo-2,8,9,10-tetrahydropyrano[2,3-f]chromen-5-yl)oxy)acetamide | 4.44E-03 |
| steviolbioside | 4.62E-03 |
| Phosphatidylcholine | 5.21E-03 |
| Colladine [M-H2O+H]+ | 5.29E-03 |
| Asulam | 6.23E-03 |
| (2S)-2-[2-[(5R,6R,7S,9S,16R,18S,19S)-19-amino-6-[(3S)-3,4-dicarboxybutanoyl]oxy-16,18-dihydroxy-5,9-dimethylicosan-7-yl]oxy-2-oxoethyl]butanedioic acid | 6.26E-03 |
| Dehydropachymic acid | 8.29E-03 |
| [3,4,5-trihydroxy-6-(3,4,5-trihydroxybenzoyl)  oxyoxan-2-yl]methyl 3,4,5-trihydroxybenzoate | 8.71E-03 |
| Propyl thiosulfate | 9.91E-03 |
| (E)-4-[(1R,2S,3S,4R,8aS)-2,3,4-trihydroxy-2,5,5,8a-tetramethyl-3,4,4a,6,7,8-hexahydro-1H-naphthalen-1-yl]but-3-en-2-one | 1.00E-02 |
| 2-amino-5-[2-[[2,3-dihydroxy-2-(1-hydroxyethyl)butanoyl]oxymethyl]-4-hydroxyanilino]-5-oxopentanoic acid | 1.08E-02 |
| Glutamine | 1.11E-02 |
| PFAP-FT_diPAP | 1.18E-02 |
| methyl 4-(4,6-dihydroxy-5-methoxy-2,5-dimethyl-3-oxocyclohexen-1  -yl)oxy-2-hydroxy-3,6-dimethylbenzoate | 1.18E-02 |
| Sorbitane Monooleate - Polysorbate 20 in-source fragment | 1.29E-02 |
| Glabrol | 1.41E-02 |
| Demethylzeylasteral | 1.48E-02 |
| (E)-1-[3-[(2,3-dihydroxyphenyl)methyl]-2,4-dihydroxy-6-methoxyphenyl]-3-phenylprop-2-en-1-one | 1.57E-02 |
| 8,8-dimethyl-2-phenylpyrano[2,3-f]chromen-4-one | 1.69E-02 |
| 5,7-Dihydroxyflavanone | 1.76E-02 |
| Alpha-Mangostin | 1.95E-02 |
| (4,8,8-trimethyldecahydro-1,4-methanoazulen-9-yl)methanol | 1.95E-02 |
| Anserine | 2.08E-02 |
| NEPSILON,NEPSILON,NEPSILON-TRIMETHYLLYSINE | 2.13E-02 |
| 7-Hydroxymitragynine | 2.23E-02 |
| Ala-Ile | 2.36E-02 |
| Soy bean phospholipid | 2.37E-02 |
| Hecogenin | 2.44E-02 |
| ginnalin A | 2.58E-02 |
| andrastin A | 2.67E-02 |
| anthothecol | 2.69E-02 |
| Swertisin | 2.83E-02 |
| (3R,7R,8R,8aS)-3,4'-dihydroxy-7'-(2-hydroxyethyl)-4,4,7,8a-tetramethylspiro[2,3,4a,5,6,7-hexahydro-1H-naphthalene-8,2'-3,8-dihydrofuro[2,3-e]isoindole]-6'-one | 2.85E-02 |
| 4-amino-2-hydroxy-5-[[1-hydroxy-1-(5-oxo-6-bicyclo[4.1.0]hept-3-enyl)propan-2-yl]amino]-5-oxopentanoic acid | 2.86E-02 |
| LEVODOPA | 2.89E-02 |
| Huperzine A | 2.92E-02 |
| Okaramine J_120151 | 3.07E-02 |
| Corynoxine | 3.21E-02 |
| Vindoline | 3.34E-02 |
| Chloratranol | 3.43E-02 |
| Cimiracemoside D | 3.51E-02 |
| 2-Hydroxychalcone | 3.53E-02 |
| 8-[4,5-dihydroxy-6-(hydroxymethyl)-3-[3,4,5-trihydroxy-6-(hydroxymethyl)oxan-2-yl]oxyoxan-2-yl]-5,7-dihydroxy-2-(4-hydroxyphenyl)chromen-4-one | 3.59E-02 |
| 5-[1,2,4a-trimethyl-5-(3-methylbutanoyloxymethyl)-2,3,4,7,8,8a-hexahydronaphthalen-1-yl]-3-methylpentanoic acid | 3.62E-02 |
| 6,3'-Dimethoxyflavone | 3.68E-02 |
| Mesoridazine | 4.06E-02 |
| Myricetin-3-O-galactoside | 4.11E-02 |
| CHOLINE | 4.16E-02 |
| PYRIDOXAMINE | 4.37E-02 |
| 3-(5-phenylthiophen-2-yl)prop-2-ynyl Acetate | 4.46E-02 |
| LPE 18:1 | 4.75E-02 |
| Oxaloacetic acid | 5.00E-02 |
| Phosphatidylcholine lyso | 5.00E-02 |
